# Supplementary material for: Pyruvate Dehydrogenase Kinase 1 inhibition mediated oxidative phosphorylation enhancement in cartilage promotes osteoarthritis progression
Source: BMC Musculoskelet Disord. 2023 Jul 20;24:597. doi: 10.1186/s12891-023-06585-6 (PMC10357736; doi:10.1186/s12891-023-06585-6)
Supplement: Supplementary file 1 — Additional file 1. [file 12891_2023_6585_MOESM1_ESM.zip › MF.Gsea.1653627316242/GOMF_PROMOTER_SPECIFIC_CHROMATIN_BINDING.html]

Details for gene set GOMF\_PROMOTER\_SPECIFIC\_CHROMATIN\_BINDING[GSEA]

|  || Dataset | 55457\_collapsed\_to\_symbols.cls.cls #OA\_versus\_normal.cls.cls #OA\_versus\_normal\_repos |
| Phenotype | cls.cls#OA\_versus\_normal\_repos |
| Upregulated in class | normal |
| GeneSet | GOMF\_PROMOTER\_SPECIFIC\_CHROMATIN\_BINDING |
| Enrichment Score (ES) | -0.36631957 |
| Normalized Enrichment Score (NES) | -1.4374645 |
| Nominal p-value | 0.033546325 |
| FDR q-value | 0.24193686 |
| FWER p-Value | 1.0 |
Table: GSEA Results Summary

  

Fig 1: Enrichment plot: GOMF\_PROMOTER\_SPECIFIC\_CHROMATIN\_BINDING      
 Profile of the Running ES Score & Positions of GeneSet Members on the Rank Ordered List

  

| SYMBOL | TITLE | RANK IN GENE LIST | RANK METRIC SCORE | RUNNING ES | CORE ENRICHMENT || 1 | NDN | "necdin, MAGE family member [Source:HGNC Symbol;Acc:HGNC:7675]" | 191 | 0.827 | 0.0285 | No |
| 2 | GTF2B | general transcription factor IIB [Source:HGNC Symbol;Acc:HGNC:4648] | 1315 | 0.455 | -0.0352 | No |
| 3 | ERCC4 | "ERCC excision repair 4, endonuclease catalytic subunit [Source:HGNC Symbol;Acc:HGNC:3436]" | 1696 | 0.400 | -0.0438 | No |
| 4 | ZNF304 | zinc finger protein 304 [Source:HGNC Symbol;Acc:HGNC:13505] | 1739 | 0.393 | -0.0265 | No |
| 5 | PCGF1 | polycomb group ring finger 1 [Source:HGNC Symbol;Acc:HGNC:17615] | 2272 | 0.329 | -0.0507 | No |
| 6 | ISL1 | ISL LIM homeobox 1 [Source:HGNC Symbol;Acc:HGNC:6132] | 2373 | 0.318 | -0.0418 | No |
| 7 | ZNF750 | zinc finger protein 750 [Source:HGNC Symbol;Acc:HGNC:25843] | 2449 | 0.308 | -0.0315 | No |
| 8 | PCGF2 | polycomb group ring finger 2 [Source:HGNC Symbol;Acc:HGNC:12929] | 2452 | 0.308 | -0.0156 | No |
| 9 | DNMT1 | DNA methyltransferase 1 [Source:HGNC Symbol;Acc:HGNC:2976] | 2836 | 0.267 | -0.0314 | No |
| 10 | ERCC1 | "ERCC excision repair 1, endonuclease non-catalytic subunit [Source:HGNC Symbol;Acc:HGNC:3433]" | 2948 | 0.255 | -0.0267 | No |
| 11 | TRIM28 | tripartite motif containing 28 [Source:HGNC Symbol;Acc:HGNC:16384] | 3526 | 0.200 | -0.0612 | No |
| 12 | SMAD3 | SMAD family member 3 [Source:HGNC Symbol;Acc:HGNC:6769] | 4061 | 0.150 | -0.0949 | No |
| 13 | GADD45A | growth arrest and DNA damage inducible alpha [Source:HGNC Symbol;Acc:HGNC:4095] | 4081 | 0.148 | -0.0887 | No |
| 14 | STAT1 | signal transducer and activator of transcription 1 [Source:HGNC Symbol;Acc:HGNC:11362] | 4495 | 0.113 | -0.1149 | No |
| 15 | SETDB1 | SET domain bifurcated histone lysine methyltransferase 1 [Source:HGNC Symbol;Acc:HGNC:10761] | 4891 | 0.080 | -0.1414 | No |
| 16 | FOXC1 | forkhead box C1 [Source:HGNC Symbol;Acc:HGNC:3800] | 5227 | 0.054 | -0.1647 | No |
| 17 | EHMT2 | euchromatic histone lysine methyltransferase 2 [Source:HGNC Symbol;Acc:HGNC:14129] | 5507 | 0.032 | -0.1848 | No |
| 18 | TP53 | tumor protein p53 [Source:HGNC Symbol;Acc:HGNC:11998] | 5511 | 0.032 | -0.1834 | No |
| 19 | MACROH2A1 | macroH2A.1 histone [Source:HGNC Symbol;Acc:HGNC:4740] | 5953 | -0.002 | -0.2176 | No |
| 20 | FOXO1 | forkhead box O1 [Source:HGNC Symbol;Acc:HGNC:3819] | 6511 | -0.043 | -0.2587 | No |
| 21 | EZH2 | enhancer of zeste 2 polycomb repressive complex 2 subunit [Source:HGNC Symbol;Acc:HGNC:3527] | 6562 | -0.047 | -0.2602 | No |
| 22 | RBL2 | RB transcriptional corepressor like 2 [Source:HGNC Symbol;Acc:HGNC:9894] | 6764 | -0.060 | -0.2727 | No |
| 23 | IKZF3 | IKAROS family zinc finger 3 [Source:HGNC Symbol;Acc:HGNC:13178] | 6905 | -0.073 | -0.2798 | No |
| 24 | POLR2A | RNA polymerase II subunit A [Source:HGNC Symbol;Acc:HGNC:9187] | 7103 | -0.088 | -0.2905 | No |
| 25 | WDR13 | WD repeat domain 13 [Source:HGNC Symbol;Acc:HGNC:14352] | 7695 | -0.134 | -0.3295 | No |
| 26 | RBL1 | RB transcriptional corepressor like 1 [Source:HGNC Symbol;Acc:HGNC:9893] | 7776 | -0.140 | -0.3284 | No |
| 27 | SOX10 | SRY-box transcription factor 10 [Source:HGNC Symbol;Acc:HGNC:11190] | 7788 | -0.141 | -0.3219 | No |
| 28 | MYOD1 | myogenic differentiation 1 [Source:HGNC Symbol;Acc:HGNC:7611] | 7839 | -0.143 | -0.3183 | No |
| 29 | HDAC1 | histone deacetylase 1 [Source:HGNC Symbol;Acc:HGNC:4852] | 7911 | -0.148 | -0.3161 | No |
| 30 | NRL | neural retina leucine zipper [Source:HGNC Symbol;Acc:HGNC:8002] | 7936 | -0.150 | -0.3101 | No |
| 31 | POU4F2 | POU class 4 homeobox 2 [Source:HGNC Symbol;Acc:HGNC:9219] | 8292 | -0.178 | -0.3284 | No |
| 32 | E2F4 | E2F transcription factor 4 [Source:HGNC Symbol;Acc:HGNC:3118] | 8445 | -0.190 | -0.3303 | No |
| 33 | ATF2 | activating transcription factor 2 [Source:HGNC Symbol;Acc:HGNC:784] | 8592 | -0.201 | -0.3312 | No |
| 34 | KDM1A | lysine demethylase 1A [Source:HGNC Symbol;Acc:HGNC:29079] | 8608 | -0.202 | -0.3218 | No |
| 35 | ERCC3 | "ERCC excision repair 3, TFIIH core complex helicase subunit [Source:HGNC Symbol;Acc:HGNC:3435]" | 8669 | -0.206 | -0.3157 | No |
| 36 | MECP2 | methyl-CpG binding protein 2 [Source:HGNC Symbol;Acc:HGNC:6990] | 8687 | -0.208 | -0.3061 | No |
| 37 | GTF2F1 | general transcription factor IIF subunit 1 [Source:HGNC Symbol;Acc:HGNC:4652] | 8910 | -0.225 | -0.3116 | No |
| 38 | SIRT1 | sirtuin 1 [Source:HGNC Symbol;Acc:HGNC:14929] | 8947 | -0.227 | -0.3025 | No |
| 39 | HDAC2 | histone deacetylase 2 [Source:HGNC Symbol;Acc:HGNC:4853] | 9359 | -0.260 | -0.3209 | No |
| 40 | HNRNPU | heterogeneous nuclear ribonucleoprotein U [Source:HGNC Symbol;Acc:HGNC:5048] | 9847 | -0.306 | -0.3428 | No |
| 41 | SUZ12 | SUZ12 polycomb repressive complex 2 subunit [Source:HGNC Symbol;Acc:HGNC:17101] | 10104 | -0.331 | -0.3454 | No |
| 42 | PRDM1 | PR/SET domain 1 [Source:HGNC Symbol;Acc:HGNC:9346] | 10374 | -0.358 | -0.3476 | Yes |
| 43 | PDX1 | pancreatic and duodenal homeobox 1 [Source:HGNC Symbol;Acc:HGNC:6107] | 10409 | -0.362 | -0.3313 | Yes |
| 44 | DHX9 | DExH-box helicase 9 [Source:HGNC Symbol;Acc:HGNC:2750] | 10627 | -0.386 | -0.3280 | Yes |
| 45 | NUP98 | nucleoporin 98 and 96 precursor [Source:HGNC Symbol;Acc:HGNC:8068] | 10875 | -0.413 | -0.3256 | Yes |
| 46 | IRF3 | interferon regulatory factor 3 [Source:HGNC Symbol;Acc:HGNC:6118] | 10879 | -0.414 | -0.3041 | Yes |
| 47 | MED1 | mediator complex subunit 1 [Source:HGNC Symbol;Acc:HGNC:9234] | 11146 | -0.450 | -0.3013 | Yes |
| 48 | HSF1 | heat shock transcription factor 1 [Source:HGNC Symbol;Acc:HGNC:5224] | 11189 | -0.456 | -0.2807 | Yes |
| 49 | GLI2 | GLI family zinc finger 2 [Source:HGNC Symbol;Acc:HGNC:4318] | 11195 | -0.456 | -0.2572 | Yes |
| 50 | BMI1 | "BMI1 proto-oncogene, polycomb ring finger [Source:HGNC Symbol;Acc:HGNC:1066]" | 11639 | -0.523 | -0.2643 | Yes |
| 51 | ATF4 | activating transcription factor 4 [Source:HGNC Symbol;Acc:HGNC:786] | 11999 | -0.593 | -0.2612 | Yes |
| 52 | EGR1 | early growth response 1 [Source:HGNC Symbol;Acc:HGNC:3238] | 12052 | -0.607 | -0.2335 | Yes |
| 53 | FOXC2 | forkhead box C2 [Source:HGNC Symbol;Acc:HGNC:3801] | 12218 | -0.650 | -0.2123 | Yes |
| 54 | FOXO4 | forkhead box O4 [Source:HGNC Symbol;Acc:HGNC:7139] | 12240 | -0.656 | -0.1796 | Yes |
| 55 | NIPBL | NIPBL cohesin loading factor [Source:HGNC Symbol;Acc:HGNC:28862] | 12385 | -0.703 | -0.1540 | Yes |
| 56 | CHD7 | chromodomain helicase DNA binding protein 7 [Source:HGNC Symbol;Acc:HGNC:20626] | 12591 | -0.791 | -0.1285 | Yes |
| 57 | ZNF609 | zinc finger protein 609 [Source:HGNC Symbol;Acc:HGNC:29003] | 12635 | -0.814 | -0.0892 | Yes |
| 58 | DDX5 | DEAD-box helicase 5 [Source:HGNC Symbol;Acc:HGNC:2746] | 12834 | -1.038 | -0.0503 | Yes |
| 59 | KLF4 | Kruppel like factor 4 [Source:HGNC Symbol;Acc:HGNC:6348] | 12848 | -1.058 | 0.0040 | Yes |
Table: GSEA details [plain text format]

  

Fig 2: GOMF\_PROMOTER\_SPECIFIC\_CHROMATIN\_BINDING      
 Blue-Pink O' Gram in the Space of the Analyzed GeneSet

  

Fig 3: GOMF\_PROMOTER\_SPECIFIC\_CHROMATIN\_BINDING: Random ES distribution      
 Gene set null distribution of ES for **GOMF\_PROMOTER\_SPECIFIC\_CHROMATIN\_BINDING**
